# PxDorsal Regulates the Expression of Antimicrobial Peptides and Affects the Bt Susceptibility of *Plutella xylostella*

**DOI:** 10.3390/insects16020163

**Published:** 2025-02-05

**Authors:** Yan Sun, Haoqi Wen, Wenrui Xue, Xiaofeng Xia

**Affiliations:** 1 State Key Laboratory of Agricultural and Forestry Biosecurity, Institute of Applied Ecology, Fujian Agriculture and Forestry University, Fuzhou 350002, China; yansun@fafu.edu.cn (Y.S.); 13338109652@163.com (H.W.); xuewenrui@fafu.edu.cn (W.X.); 2 Key Laboratory of Integrated Pest Management for Fujian-Taiwan Crops, Ministry of Agriculture and Rural Affairs, Fuzhou 350002, China; 3 Joint International Research Laboratory of Ecological Pest Control, Ministry of Education, Fuzhou 350002, China; 4 Youxi-Yangzhong Vegetable Pest Prevention and Control, Fujian Observation and Research Station, Fuzhou 350002, China

**Keywords:** *Plutella xylostella*, Toll pathway, NF-κB pathway, *Dorsal*, *Cactus*, antimicrobial peptides

## Abstract

The insect NF-κB pathway is primarily constituted by nuclear factor κB (NF-κB) and the inhibitor of κB (IκB), which plays a crucial role in the innate immune response. Dorsal and Cactus, as NF-κB and IκB factors, are important downstream regulators of the Toll pathway in *Plutella xylostella*. In this study, the *PxDorsal* and *PxCactus* genes of *P. xylostella* were cloned, and the molecular docking demonstrated that PxDorsal and PxCactus can interact with each other. RT-qPCR results indicated that *PxDorsal* and *PxCactus* were expressed in all stages, and the expression of *PxDorsal*, *PxCactus*, and antimicrobial peptides *PxGloverin*2, *PxMoricin*3, and *PxLysozyme*2 were significantly down-regulated under *Bacillus thuringiensis* (Bt8010) infection. Interestingly, silencing the *PxDorsal* gene by RNA interference (RNAi) significantly down-regulated the expression of *PxGloverin*2 and *PxMoricin*3 and increased the epidermis melanization of *P. xylostella* larvae fed with Bt8010. Our findings indicate that PxDorsal and PxCactus may interact with each other, and silencing *PxDorsal* inhibits the expression of downstream antimicrobial peptides, thereby enhance the susceptibility of *P. xylostella* to Bt8010. This study contributes a theoretical basis for further research on the Toll pathway of *P. xylostella* to pathogens and offers insights for screening effective biological control targets from the perspective of the immune system.

## Simple Summary

Insects living in natural environments are often infected by various pathogens, and they require a strong immune system. The Toll pathway is an important pathway in the insect innate immune system that helps insects resist pathogens. Our study investigated how *PxDorsal* regulates the expression of antimicrobial peptides in response to Bt8010 in the Toll signaling pathway. *PxDorsal* and *PxCactus* were cloned, and their interaction was confirmed by molecular docking. Silencing *PxDorsal* by RNAi down-regulated the expression of antimicrobial peptides *PxGloverin*2 and *PxMoricin*3, and significantly increased the epidermis melanization of *Plutella xylostella* larvae to Bt8010 infection. Our study identified a possible interaction between PxDorsal and PxCactus and demonstrated that *PxGloverin*2 and *PxMoricin*3 are regulated by the Toll pathway in *P. xylostella*. The current research revealed the correlation between the insect immune system and Bt susceptibility, providing insights for future pest biological control based on immunity.

## 1. Introduction

The Toll pathway is one of the insect immune pathways that regulates the expression of antimicrobial peptides. The insect NF-κB pathway is composed of nuclear factor κB (NF-κB) and the inhibitor of κB (IκB) and is the key to the immune response [1]. NF-κB contains an RHD (N-terminal Rel homology domain) and an IPT (immunoglobulin-plexin-transcription domain). The RHD contains a target gene binding region, a nuclear localization signal region, and an IκB binding region [2,3,4]. There are three NF-κB proteins in insects, namely Dorsal, Dif, and Relish. IκB proteins contain three typical structural features: five to seven ankyrin repeats (ANKs), a degradation structural unit sequence (DSGFLS), and PEST functional domain. The number of ANKs may vary among species, for instance, the *Drosophila melanogaster*, *Bombyx mori*, *Plutella xylostella*, and *Scylla paramamosain* contain five ANK sequences [5,6,7], and *Cyclina sinensis* and *Fenneropenaeus chinensis* contain six ANK sequences [8]. NF-κB binds to IκB to form a complex and inhibits nuclear translocation [9,10]. Specially, *D. melanogaster*’s DmCactus, a homologue of IκB, binds to DmDorsal [5], and LvCactus from *Litopenaeus vannamei* interacts with LvDorsal and prevents nuclear translocation [9]. Moreover, numerous studies have confirmed that NF-κB proteins are essential in host immune defense against pathogenic microorganisms [2,3,11,12,13,14,15]. Upon stimulation by fungi and Gram-positive bacteria, the host activates the Toll pathway, triggering the phosphorylation of Pelle and the disruption of Cactus, inducing the release of Dif/Dorsal into the nucleus, thereby activating the expression of antimicrobial peptide genes [1,4,14,15,16,17,18,19,20]. Our previous study has shown that the genome of *P. xylostella* also contains a complete Toll pathway [21], and further studies found that the Toll pathway in the gut of *P. xylostella* can be activated by both Gram-negative bacteria and Gram-positive bacteria [22].

The diamondback moth *P*. *xylostella* (L.) (Lepidoptera: Plutellidae), is one of the most prominent pests of Brassicaceae crops [23]. The inappropriate use of insecticides has resulted in an increased resistance in *P. xylostella*, even to the biological pesticide Bt [24]. In several insect species, such as *Galleria mellonella*, *Tribolium castaneum*, *B. mori*, *Helicoverpa armigera*, and *Spodoptera litura*, Bt or Bt toxins have been found to activate the host immune defense system [25,26,27,28,29,30]. In *P. xylostella*, Dorsal in the midgut digestive fluid was found to bind to Cry1Ab1, thereby affecting the host’s Bt susceptibility [31]. Additionally, silencing the *Dorsal* gene down-regulates the *Defensin* expression and increases the insecticidal activity of Bt (HD73) in *P. xylostella* [32]. Another study also revealed that silencing the *Cactus* gene in *P. xylostella* for 24 h significantly up-regulates the expression of antimicrobial peptide genes [33]. These findings indicate that the insect innate immune system plays a crucial role in defending against pathogenic invasions. Understanding the molecular mechanisms behind the host’s immune response to Bt infection is essential for advancing biological pest control strategies.

The detailed functional analysis of the NF-κB pathway has been performed in *Drosophila* and *B. mori* [1,3,7,34,35], and even some studies have been performed in *P. xylostella*; however, do the PxDorsal and PxCactus in the *P. xylostella* interact with each other? What is the structural morphology of their interaction? Which antimicrobial peptides are regulated by the interaction of these two proteins? Is the variation in these antimicrobial peptides related to the susceptibility of the *P. xylostella* to Bt? These issues are still not clear enough at present. Therefore, to address these issues, the present study cloned the *PxDorsal* and *PxCactus* genes, performed molecular docking, and detected their expression at different stages. Additionally, this study analyzed the expression changes in the downstream immune genes of the Toll pathway after Bt8010 feeding, and RNA interference (RNAi) was utilized to knock down the *PxDorsal* gene to study its function in vivo. Overall, this study aims to comprehensively understand the interaction of PxDorsal and PxCactus, which regulates the expression of antimicrobial peptides and affects the Bt susceptibility of *P. xylostella* and provides insights for finding new control targets.

## 2. Materials and Methods

### 2.1. Test Strains and Plasmids

*B. thuringiensis* strain Bt8010 (FJIAE-Bt8010) was donated by Prof. Guan X. of Fujian Agriculture and Forestry University. *Escherichia coli* DH5α (FJIAE-DH5α) and pET-28a-*EGFP* (FJIAE-*EGFP*) strains were purchased and preserved in the Institute of Applied Ecology, Fujian Agriculture and Forestry University.

### 2.2. Test Insects

*P. xylostella* is reared in the laboratory using an artificial diet. Culture conditions in the artificial climate chamber are maintained at 25 ± 1 °C, in a 16:8 h light–dark photoperiod, and 70–80% relative humidity. The artificial diet was slightly modified on the basis of the previous laboratory formula [36], adding 25 μL linoleic acid to the artificial diet. Linoleic acid plays an important role in promoting growth and improving the feed palatability of *P. xylostella*. Finally, the mixture is poured into boxes, with 70 mL per box, and allowed to dry at room temperature. The adults were fed with 10% honey solution.

### 2.3. Cloning of PxDorsal and PxCactus Genes

The primer sequences for the *PxDorsal* and *PxCactus* genes were designed by the DBM genome database (http://59.79.254.1/DBM/; accessed on 24 August 2021) and synthesized by the SunYa Biotechnology Company (Fuzhou, China). The primer sequences are as follows: *PxDorsal*-F (5′-3′): CCTTGGACCAGTGGAGAGAGACAGTT, *PxDorsal*-R (5′-3′): TCAACAAGTCCCCGTAGACAGTCT, *PxCactus*-F (5′-3′): ATGAGTTTCAAGAAGGATTTCGAC, and *PxCactus*-R (5′-3′): TCAGGCGACGTTGATGGCGTTCAT. The *P. xylostella* RNA was extracted using the RNA Extraction Kit (Promega, Shanghai, China). cDNA was synthesized using HiScript III All-in-one RT SuperMix Perfect Kit (Vazyme, Nanjing, China). The reaction system was as follows: RNA 2000 ng, 5× All-in-one qRT SuperMix 4.0 μL, Enzyme Mix 1.0 μL, Nuclease-free Water to 20.0 μL. The operating program was as follows: 50 °C for 15 min, 85 °C for 5 s. Using the cDNA as a template, the PCR amplification system was as follows: cDNA 1.0 μL, *PxDorsal*/*PxCactus*-F 1.0 μL, *PxDorsal*/*PxCactus*-R 1.0 μL, Phanta Mix Super-Fidelity DNA Polymerase 0.5 μL, dNTP Mix 0.5 μL, 2× Phanta Mix Buffer 12.5 μL, and Nuclease-free Water 8.5 μL. The PCR procedure was as follows: 95 °C for 3 min, 35 cycles at 95 °C—15 s, 58 °C—15 s, 72 °C—2 min, and 72 °C—5 min. The products were analyzed by 2% agarose gel electrophoresis and purified using the Gel Extraction Kit (Omega Bio-Tek, Norcross, GA, USA). Ligation was carried out using the Hieff Clone^®^ Zero TOPO-Blunt Cloning Kit (Yeasen, Shanghai, China): 10× Enhancer 1 μL, pESI-Blunt vector 1 μL, target DNA 200 ng, Nuclease-free Water added to 10 μL. The ligation products were transformed into the *E. coli* DH5α competent cell, coated in LB plates, and incubated overnight at 37 °C. The positive colonies containing pESI-PxDorsal and pESI-PxCactus plasmids were screened, and PCR was performed with M13F and M13R primers. The colony PCR system and amplification were consistent with those described above. The products were sequenced and analyzed using SnapGene 6.02 to determine the coding sequence (CDS) of *PxDorsal* and *PxCactus* genes.

### 2.4. Sequence and Structural Model Analysis of PxDorsal and PxCactus

Expasy-Protparam (https://web.expasy.org/protparam/; accessed on 10 December 2022), SignalP (https://services.healthtech.dtu.dk/services/SignalP-5.0/; accessed on 10 December 2022), TMHMM (http://www.cbs.dtu.dk/services/TMHMM/; accessed on 10 December 2022), Expasy-Hydropath (https://web.expasy.org/protscale/; accessed on 10 December 2022), NCBI (https://www.ncbi.nlm.nih.gov/Structure/cdd/wrpsb.cgi; accessed on 10 December 2022), SMART (http://smart.embl-heidelberg.de/; accessed on 10 December 2022), UniProt (https://www.uniprot.org/; accessed on 10 December 2022), NetPhos (https://services.healthtech.dtu.dk/services/NetPhos-3.1/; accessed on 10 December 2022), and Epestfind (https://www.bioinformatics.nl/cgi-bin/emboss/epestfind; accessed on 10 December 2022) were used to predict protein physicochemical properties, signal peptide, transmembrane region, hydrophilic hydrophobicity, protein function, conserved domain, subcellular localization, phosphorylation site, and PEST region.

Multiple sequences alignment was performed using ClustalW in MEGA 5.1 and the comparison results were visualized by ESPript 3.0 (https://espript.ibcp.fr/ESPript/ESPript/; accessed on 10 December 2022), and the phylogenetic tree was constructed using the neighbor-joining (N-J) algorithm of MEGA 5.1 with 1000 bootstrap replicates [37,38].

SOMPA (https://npsa-pbil.ibcp.fr/cgi-bin/npsa_automat.pl?page=npsa_sopma.html; accessed on 10 December 2022) was used to analyze the protein secondary structure, using the SWISS-MODEL (https://swissmodel.expasy.org/; accessed on 16 March 2023) to construct the protein 3-dimention model, and the homology model was evaluated by the PROCHECK test using UCLA-DOE LAB-SAVES (https://saves.mbi.ucla.edu/; accessed on 16 March 2023). Molecular docking was performed using the protein–protein docking (Piper) module in Schrödinger. The standard docking protocol was selected, with the number of rotatable bonds of the ligand set to the default maximum and the number of generated conformational states defined as 10. Following docking, energy minimization was performed to refine the interactions and ensure that a comprehensive conformational ensemble of the ligand was captured. The first-ranked conformation was selected for visualization with PyMOL.

### 2.5. Spatio-Temporal Expression Detection of PxDorsal and PxCactus Genes in P. xylostella and the Immune Genes After Bt Infection

The eggs of *P. xylostella* (each tube covered the bottom of a 1.5 mL centrifuge tube), were collected alongside 30 1st instar larvae, 20 2nd instar larvae, 10 3rd instar larvae, 5 4th instar larvae, 5 pupae, and 5 adults, and 3 tubes were collected each instar. Then, we extracted the RNA from all the samples, respectively, and reverse transcribed it into cDNA, and stored it at −20 °C for later use.

*B. thuringiensis* Bt8010 glycerol strain stored at −80 °C was inoculated into 100 mL of liquid LB medium and activated at 30 °C 180 rpm for 10 h. Subsequently, 10 mL of the activated culture was inoculated into 1 L of liquid LB and further incubated at 30 °C 180 rpm for 10 h. We centrifuged the mixture at 4 °C, 6000 rpm for 10 min to collect the precipitate. After cleaning the precipitate three times with sterile water, we centrifuged again. Finally, a little sterile water was added and mixed to measure the optical density at 600 nm (OD_600_). The normal artificial diet was prepared, and the following components were added: Bt8010-treated diet (Bt8010): Bt8010 was added to achieve the total diet concentration reach OD_600_ = 2.3; control diet (CK): An equal volume of sterile water was added. The 4th instar larvae of *P. xylostella*, exhibiting uniform growth status, were selected for the experiment, with 15 larvae per group. After 4 h of starvation, they were fed with two different artificial diets, with 3 replicates in each group. Following 12 h of feeding, larvae were collected (5 per treatment), and all samples were quickly frozen to extract RNA which was reverse transcribed into cDNA and stored at −20 °C for later use. The *P. xylostella* ribosomal protein *RPL*32 gene was selected as an internal reference for RT-qPCR primers, and additional RT-qPCR primers (Table S1, Figure S1A–G) were designed and sent to SunYa Biotechnology Company (Fuzhou, China) for synthesis. RT-qPCR reactions were conducted using the Taq Pro Universal SYBR qPCR Master Mix (Vazyme, Nanjing, China). The amplification system was as follows: cDNA 1.5 μL, *PxDorsal*/*PxCactus*/*PxGloverin*2/*PxMoricin*3/*PxCecropin*2/*PxLysozyme*2/*RPL*32-F 0.4 μL, *PxDorsal*/*PxCactus*/*PxGloverin*2/*PxMoricin*3/*PxCecropin*2/*PxLysozyme*2/*RPL*32-R 0.4 μL, 2× Taq Pro Universal SYBR qPCR Master Mix 10 μL, and Nuclease-free Water 7.7 μL. The procedure was as follows: 95 °C for 30 s, 40 cycles at 95 °C—10 s, 60 °C—30 s. The 2^−ΔΔCT^ method [39] was used to analyze the relative expression of immune genes.

### 2.6. RNAi of PxDorsal Gene and Its Effects on the Expression of Antimicrobial Peptides of P. xylostella

RNAi primers (Table S2) targeting the functional domain of *PxDorsal* using NCBI Primer-BLAST (https://www.ncbi.nlm.nih.gov/tools/primer-blast/index.cgi?LINK_LOC=BlastHome; accessed on 24 October 2021) were designed and sent to SunYa Biotechnology Company (Fuzhou, China) for synthesis. PCR amplification was conducted using pESI-PxDorsal and EGFP plasmids as templates, and ds*PxDorsal*/ds*EGFP*-F and ds*PxDorsal*/ds*EGFP*-R were used as primers, and the amplification system and purification were described in step 2.3. The T7RiboMAX^TM^ Express RNAi System (Promega, Beijing, China) was used to synthesize ds*PxDorsal* and ds*EGFP* double-stranded RNA in vitro. The reverse transcription system was as follows: RiboMAX^TM^ Express T7 2× Buffer 40.0 μL, Enzyme Mix T7 Express 8.0 μL, DNA template 8.0 Μl, and Nuclease-free Water 24.0 μL. After incubation at 37 °C for 4 h, 1 μL DNase stock solution and 1 μL 0.5% RNase diluent (RNase stock solution was diluted 200 times in advance) were added, and the incubation was continued for 30 min. The synthesized ds*PxDorsal* and ds*EGFP* were subjected to RNA purification, and the purification steps were shown in the T7RiboMAXTM Express RNAi System (Promega, Beijing, China). It was found in RNAi studies in *Drosophila* that dsRNA and Lipofectamine can promote dsRNA interference after coincubation to form a complex [40]. The quantified 900 ng of ds*PxDorsal*, 1500 ng of ds*PxDorsal*, and 900 ng of (ds*PxDorsal* + Lipofectamine mixture) (dsRNA/Lipofectamine (*w*/*v*) was 5:1) were injected into the 4th instar larvae of *P. xylostella* using a handheld injector (Drummond, Germany), and the ds*EGFP* as the control group. After 12 h of injection, 5 test insects were collected as one biological repeat and three biological repeats were conducted. The samples were collected and immediately frozen to extract the RNA, which was reverse-transcribed into cDNA for RT-qPCR analysis to detect the interference efficiency of dsRNA injection for 12 h. The 4th instar larvae of *P. xylostella* were collected 12 h after dsRNA (ds*PxDorsal*/ds*EGFP* + Lipofectamine) injection and quickly frozen to extract RNA, which was reverse-transcribed into cDNA for the RT-qPCR analysis of the downstream antimicrobial peptides (*PxGloverin*2, *PxMoricin*3, *PxCecropin*2, *PxLysozyme*2) of the *PxDorsal* gene. The interference efficiency and expression levels were calculated using the 2^−ΔΔCT^ method [39].

### 2.7. The Epidermis Melanization of P. xylostella Under the Infection of Bt8010

RNAi was conducted on the 4th instar larvae of *P. xylostella*, utilizing dsRNA (ds*PxDorsal* + Lipofectamine) at a concentration of 900 ng as the experimental group, and ds*EGFP* as the control group. A total of 60 test insects were included in each treatment group, with 20 insects per biological replicate and three biological replicates in total. Immediately after RNA injection, the larvae were fed with a Bt8010 diet, and the epidermis melanization of *P. xylostella* was counted 12 h later.

### 2.8. Statistical Analysis

IBM SPSS Statistics 22 was used to perform an LSD test in one-way ANOVA for the expression level data of different instars, and sqrt function was used to transform the expression levels of immune genes, and Student’s *t* test was used for analysis. The data of the gene expression and melanization rate after RNAi were analyzed by Student’s *t* test. Data were performed with mean ± standard error (SEM). GraphPad Prism 9 was used for plotting.

## 3. Results

### 3.1. Cloning and Sequence Analysis of PxDorsal and PxCactus

The CDS sequences of the *Px0008539* and *Px0016665* genes were cloned from *P. xylostella* by PCR and uploaded to GenBank as *PxDorsal* (GenBank: OQ439920.1) and *PxCactus* (GenBank: OQ439953.1). The open reading frame (ORF) of the *PxDorsal* gene is 1656 bp, encoding 551 amino acid residues (Figure S2A), with an RHD conserved domain (17—187 amino acid residues), an IPT conserved domain (192—291 amino acid residues). The molecular formula is C_2648_H_4173_N_765_O_788_S_40_, and its theoretical molecular weight is 60.62 KDa. Proline (Pro) is the most abundant amino acid (11.4%), and Tryptophan (Trp) is the least (0.5%) (Table S3). The calculated isoelectric point (pI) is 7.66. PxDorsal is hydrophilic, weakly alkaline, lacks a signal peptide, and is an unstable protein, mainly located in the cytoplasm (Figure S2B,C). The *PxCactus* ORF is 1053 bp, encoding 350 amino acid residues (Figure S3A), with 5 consecutive ANK conserved domains (114—290 amino acids). The molecular formula is C_1610_H_2561_N_453_O_547_S_16_, and the theoretical molecular weight is 37.53 KDa. Alanine (Ala) is the most prevalent amino acid (11.4%), while Tryptophan (Trp) is the least (0.6%) (Table S4), and the calculated pI is 4.48. PxCactus is predicted to lack a signal peptide and is an acidic, hydrophilic, and unstable protein primarily localized in the cytoplasm (Figure S3B,C). These results show that PxDorsal and PxCactus were successfully cloned and analyzed, revealing them as cytoplasmic unstable proteins with specific conserved domains.

### 3.2. Sequence Alignment and Phylogenetic Analysis of PxDorsal and PxCactus

**Figure 1:**
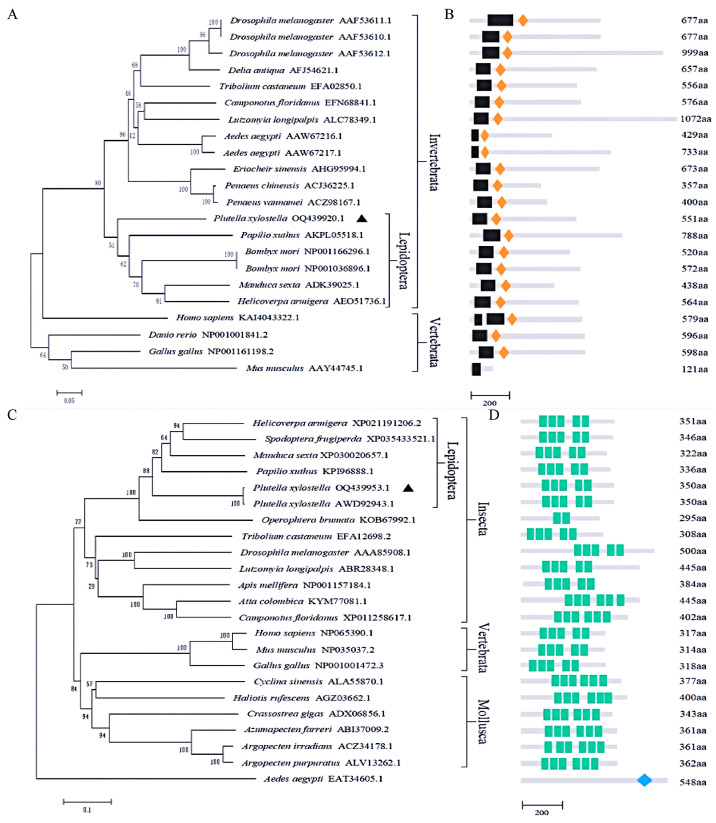
Phylogenetic analysis of NF-κB and IκB factors in different species. (**A**) Phylogenetic tree of NF-κB factors in different species, and PxDorsal is specifically marked with ▲; (**B**) NF-κB factors domain in different species; RHD domain is represented by black boxes and the orange diamond boxes represent IPT domain; (**C**) Phylogenetic tree of IκB factors of different species, and PxCactus is indicated with ▲; (**D**) IκB inhibitors domain of different species; the green boxes represent the ANKs domain, and the CactinC domain is represented by a blue diamond box.

Multiple sequence alignment revealed that PxDorsal exhibits key features of NF-κB proteins, including the typical RHD and IPT domains, 6 conserved cysteine residues, and 12 potential phosphorylation sites (Figure S4). Similarly, PxCactus displays hallmark characteristics of IκB proteins, featuring 5 ANKs, including 2 Cys residues and 11 potential phosphorylation sites (Figure S5). Phylogenetic analysis showed that NF-κB proteins are generally conserved across species. PxDorsal from *P. xylostella* clustered with Dorsal proteins from other Lepidoptera, such as *Papilio xuthus*, *B. mori*, *Manduca sexta*, and *H. armigera*, forming a distinct clade with other invertebrates like *D. melanogaster* (Figure 1A,B). In vertebrates, except for humans, NF-κB proteins were grouped together. Regarding IκB proteins, they also show relative evolutionary conservation, with insect IκB proteins clustering together. Among them, PxCactus clustered with *H. armigera*, *Spodoptera frugiperda*, *M. sexta*, *P. xuthus*, and *Operophtera brumata*, while vertebrate and mollusk IκB proteins formed separate groups (Figure 1C,D). Based on the results of the phylogenetic tree, invertebrates and vertebrates are branched into two distinct lineages, indicating a divergence among different taxa on the evolutionary tree. Moreover, within the class Insecta of invertebrates, insects from different orders have undergone remarkable order-based evolution. These differences are consistent with the functional differentiations among their corresponding species. These findings highlight that PxDorsal and PxCactus exhibit conserved features of NF-κB and IκB proteins, respectively. Phylogenetic analysis reveals the evolutionary conservation and distinct clustering among species, suggesting that the evolution of immune genes is concurrent with that of species.

### 3.3. Structural Modeling and PxDorsal–PxCactus Interaction Analysis

**Figure 2:**
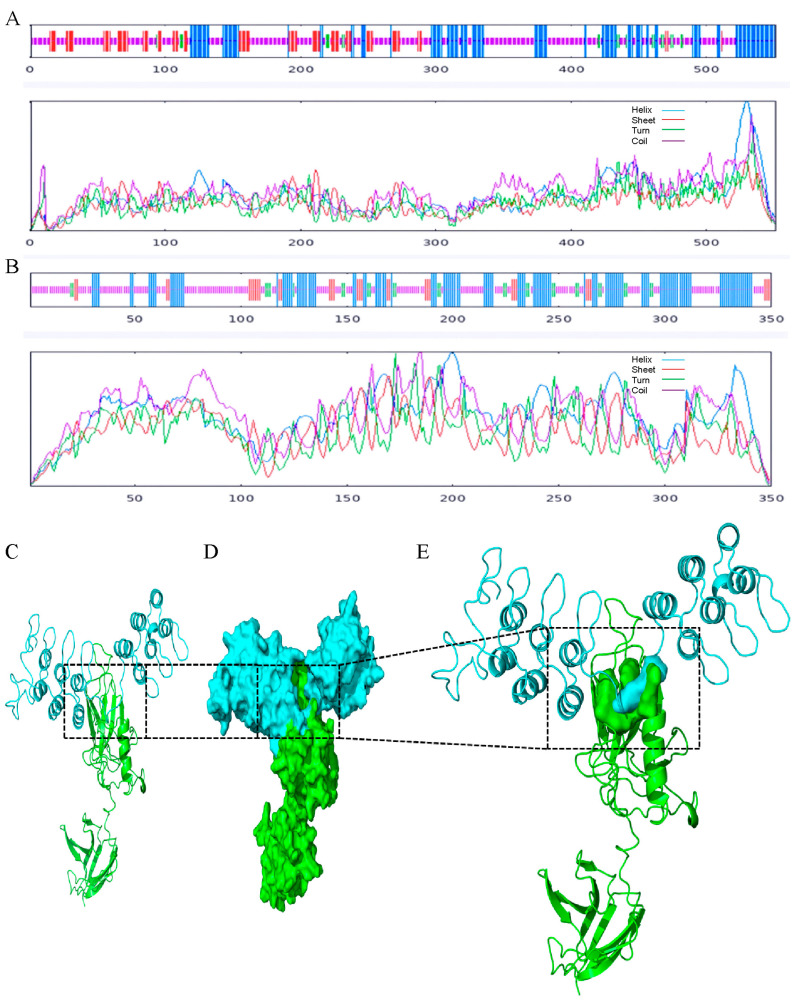
Structural analysis and protein docking of PxDorsal and PxCactus. (**A**,**B**) The secondary structures of PxDorsal (**A**) and PxCactus (**B**); (**C**) cartoon of PxDorsal and PxCactus model docking; (**D**) surface of PxDorsal and PxCactus model docking; (**E**) surface of the interacting amino acids. The green and red models represent PxDorsal and PxCactus, respectively, and they act as a key and a lock interacting with each other; interaction regions are indicated by the black box.

SOMPA analysis showed that the secondary structure of PxDorsal comprises 25.59% α-helix, 3.99% β-turn, 54.81% random coil, and 15.61% extended sheet (Figure 2A), indicating that PxDorsal primarily consists of random coils and α-helices. In contrast, the secondary structure of PxCactus contains 34.00% α-helix, 8.00% β-turn, 48.57% random coil, and 9.43% extended sheet (Figure 2B), with random coils and α-helices as the dominant structural elements.

Homologous models of PxDorsal and PxCactus were constructed using the SWISS-MODEL. The PROCHECK evaluation showed that the quality of the two models was better (Table S5). PyMOL clearly displayed the RHD and IPT domains of PxDorsal proteins and the ANKs domain of PxCactus proteins (Figure S6). The docking results by molecular simulation indicated that PxDorsal and PxCactus acted as a key and a lock, respectively, and could combine tightly together, which is the “Lock-and-Key Interaction Model” (Figure 2C–E). Further, the interaction of the amino acid binding region preliminarily confirmed that the RHD domain of PxDorsal combined with the ANKs domain of PxCactus (Figure 2E) and could combine through the hydrogen bonding between the amino acid residues (Figure S7). Particularly, the shortest hydrogen bond distance was observed between the N-terminal Arg^134^ of PxDorsal and the N-terminal Arg^213^ of PxCactus as 0.6 Å, which indicated the strongest interaction between these residues (Table S6) and facilitated the stable binding of the model. Taken together, molecular docking provides a preliminary indication that PxDorsal and PxCactus interact through stable hydrogen bonding, following the “Lock-and-Key Interaction Model”.

### 3.4. Spatio-Temporal Expression Analysis of PxDorsal and PxCactus

**Figure 3:**
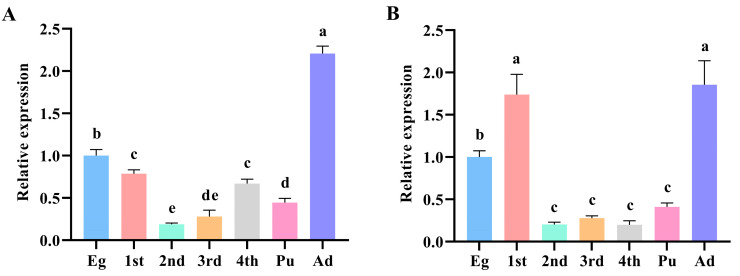
Spatio-temporal distribution of *PxDorsal* and *PxCactus* in different developmental stages of *Plutella xylostella*. *PxDorsal* (**A**) and *PxCactus* (**B**) were expressed in all instars of *P. xylostella*; Eg: egg, 1st: 1st instar larvae, 2nd: 2nd instar larvae, 3rd: 3rd instar larvae, 4th: 4th instar larvae, Pu: pupal, and Ad: adult; the data represented the mean ± SEM (*n* = 3). Different letters indicate significant differences between different stages (LSD test, *p* < 0.05).

RT-qPCR results indicated that *PxDorsal* and *PxCactus* were expressed at all developmental stages of *P. xylostella*, although their expression levels differed, and their expression trends were roughly similar (Figure 3). For *PxDorsal*, the highest expression was found in the adult stage, which was significantly higher than the other stages (*F*_6,21_ = 125.061, *p* = 0.000), and it was followed by the egg stage (*F*_6,21_ = 125.061, *p* < 0.022) (Figure 3A). Regarding *PxCactus*, its expressions in the 1st instar and adult stages were significantly higher than in the other instars (*F*_6,21_ = 24.733, *p* < 0.002), but there was no significant difference in expression between the two stages (*F*_6,21_ = 24.733, *p* = 0.579) (Figure 3B). Next, the egg stage showed significantly higher expression than the 2nd, 3rd, 4th instars, and pupal stages (*F*_6,21_ = 24.733, *p* < 0.01). In summary, *PxDorsal* and *PxCactus* are expressed across all developmental stages of *P. xylostella* with different expression levels.

### 3.5. Bt8010 Regulates the Expression of Immune Genes in P. xylostella

**Figure 4:**
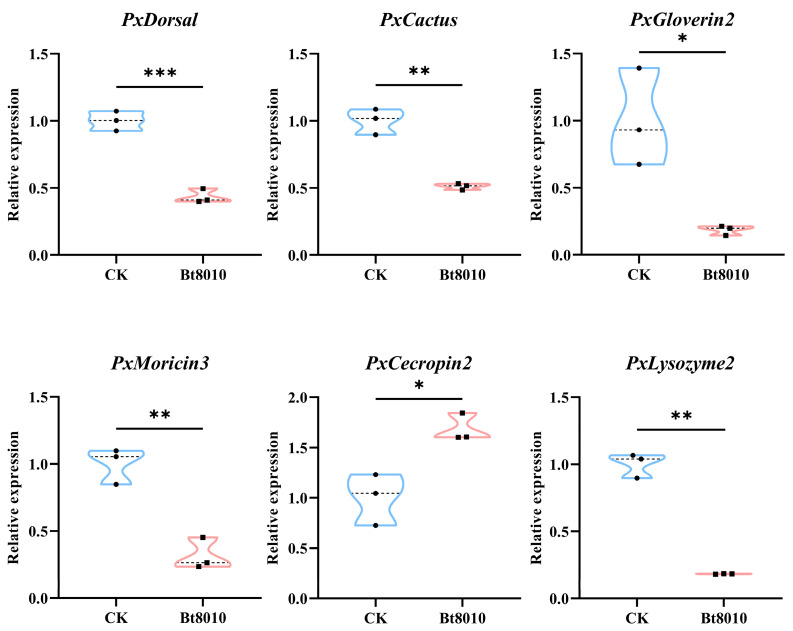
Effects of feeding Bt8010 on the expression of immune genes in *P*. *xylostella*. When *P. xylostella* fed with Bt8010, the expression of immune genes *PxDorsal* and *PxCactus* and antimicrobial peptides *PxGloverin*2, *PxMoricin*3, and *PxLysozyme*2 were significantly down-regulated; however, the expression of *PxCecropin2* was up-regulated. The data represented the mean ± SEM (*n* = 3). Student’s *t* test analysis, * *p* < 0.05, ** *p* < 0.01, and *** *p* < 0.001.

To assess the expression of target genes *PxDorsal* and *PxCactus* and antimicrobial peptides (*PxGloverin*2, *PxMoricin*3, *PxCecropin*2, *PxLysozyme*2) following Bt8010 infection, RT-qPCR was conducted. The results indicated that the expression of *PxDorsal* (*t* = 10.837, *df* = 4, *p* = 0.000), *PxCactus* (*t* = 8.547, *df* = 4, *p* = 0.001), *PxGloverin*2 (*t* = 3.865, *df* = 4, *p* = 0.018), *PxMoricin*3 (*t* = 6.654, *df* = 4, *p* = 0.003), and *PxLysozyme*2 (*t* = 15.609, *df* = 4, *p* = 0.004) were significantly down-regulated in *P. xylostella* fed with Bt8010 compared to the control group. In contrast, the expression of *PxCecropin*2 (*t* = −4.072, *df* = 4, *p* = 0.015) was significantly up-regulated (Figure 4). Overall, Bt8010 infection significantly down-regulated the expression of *PxDorsal* and *PxCactus* and most antimicrobial peptide genes in *P. xylostella*, while up-regulating *PxCecropin*2.

### 3.6. Knockdown of PxDorsal Inhibited the Expression of Antimicrobial Peptides and Increased the Epidermis Melanization Rate of P. xylostella

**Figure 5:**
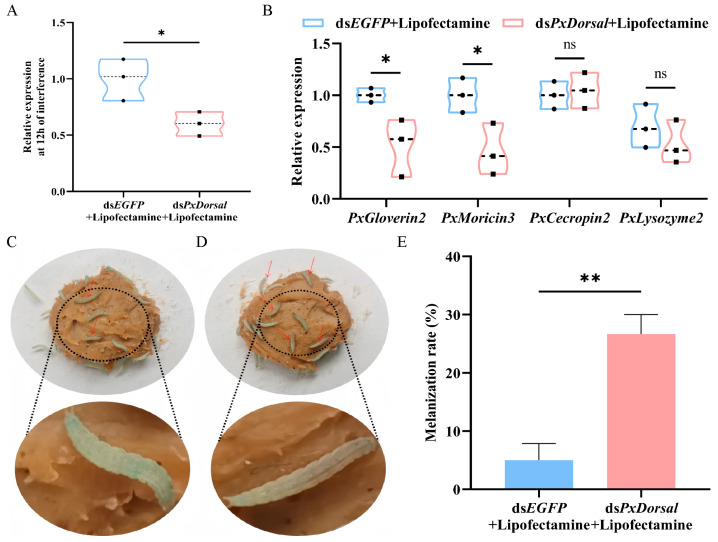
*PxDorsal* gene silencing inhibited antimicrobial peptides expression and promoted epidermis melanization in *P*. *xylostella* larvae fed with Bt8010. (**A**) The expression of the *PxDorsal* gene was significantly down-regulated after dsRNA (ds*PxDorsal*/ds*EFGP* + Lipofectamine) injection for 12 h; (**B**) the antimicrobial peptides *PxGloverin*2 and *PxMoricin*3 were significantly down-regulated after *PxDorsal* gene silencing. The data represented the mean ± SEM (*n* = 3). Student’s *t* test analysis, * *p* < 0.05, ** *p* < 0.01, and ns indicated that there was no significant difference between different treatments; (**C**) partially epidermis melanization in *P. xylostella* injected with ds*EGFP* and simultaneously fed Bt8010; (**D**) *P. xylostella* injected with ds*PxDorsal* and fed with Bt8010 showed accelerated epidermis melanization from the head. The red arrow points out the larvae of *P. xylostella* with obvious melanization of the epidermis, and the black dotted line shows the enlarged figure of the larval phenotype; (**E**) *PxDorsal* gene silencing significantly increased the melanization rate of *P. xylostella* larvae fed with Bt8010.

In order to select the optimal interference efficiency, we chose to quantify 900 ng of dsRNA (ds*PxDorsal*/ds*EGFP*), 1500 ng of dsRNA (ds*PxDorsal*/ds*EGFP*), and 900 ng of dsRNA (ds*PxDorsal*/ds*EGFP* + Lipofectamine), three groups injected into the 4th instar larvae of *P. xylostella*, and the results indicated that the target gene *PxDorsal* was significantly silenced after the injection of 1500 ng of ds*PxDorsal* (*t* = 4.563, *df* = 4, *p* = 0.01) and 900 ng of ds*PxDorsal* + Lipofectamine (*t* = 3.705, *df* = 4, *p* = 0.021) for 12 h (Figure 5A and Figure S1H). Since a high concentration of dsRNA may affect the growth and survival of *P. xylostella*, and dsRNA mixed with Lipofectamine can improve the interference efficiency, we subsequently chose to conduct the experiment by injecting 900 ng of dsRNA + Lipofectamine. Following the silencing of the *PxDorsal* gene, the expression of *PxGloverin*2 (*t* = 2.916, *df* = 4, *p* = 0.043) and *PxMoricin*3 (*t* = 2.828, *df* = 4, *p* = 0.047) were significantly down-regulated compared to the control group (ds*EGFP*), while no significant differences were observed in the expression of *PxCecropin*2 (*t* = −1.055, *df* = 4, *p* = 0.351) and *PxLysozyme*2 (*t* = 0.973, *df* = 4, *p* = 0.386) (Figure 5B). Subsequently, a morphological observation experiment showed that *PxDorsal* was silenced and simultaneously feeding Bt8010, and the melanization of the larvae epidermis was observed after 12 h (Figure 5C,D). Further statistical analysis revealed that the melanization rate in these larvae was significantly higher than the control group (*t* = −5.50, *df* = 4, *p* = 0.005) (Figure 5E). These findings suggest that *PxDorsal* regulates antimicrobial peptide expression in *P. xylostella*, playing a key role in immune response modulation during pathogen infection.

## 4. Discussion

The NF-κB pathway plays a crucial role in insect immunity and development, primarily involving NF-κB and IκB factors. In *Drosophila*, studies have shown that without pathogen invasion, NF-κB binds to IκB to form a complex, resulting in the nuclear localization signal (NLS) being blocked [41], which prevents NF-κB from entering the nucleus and fails to activate the transcription of antimicrobial peptide genes. When infected by pathogens, IκB proteins are phosphorylated and degraded by IκB kinase (IKK), leading to the dissociation of NF-κB from the complex [3,42]. This exposes the NLS of NF-κB, allowing its translocation into the nucleus and the activation of the downstream antimicrobial peptide genes [43].

Recently, Dorsal and Cactus genes have been identified in several insect and invertebrate species [13], and although some studies have been conducted on that gene in *P. xylostella*, the exact mechanism remains unclear. Therefore, in this study, the CDS of *Dorsal* and *Cactus* were successfully cloned from *P. xylostella* and named as *PxDorsal* and *PxCactus*. Phylogenetic analysis clearly demonstrated the evolutionary conservation of PxDorsal and PxCactus, but future study can focus on the elucidating of the functional correlation between functional differentiation and host evolution. Molecular docking results confirmed that PxDorsal and PxCactus proteins could interact, with the RHD domain of PxDorsal binding to the ANKs domain of PxCactus. This result is consistent with the interaction between SpCactus and SpDorsal in *S. paramamosain* and the combination of the RHD domain of the NF-κB protein with the ANKs domain of the IκB protein in *Drosophila* [13,44]. This interaction may involve signaling pathways; nevertheless, the interaction between Dorsal and Cactus in this study is solely demonstrated theoretically, and further verification by other protein–protein interaction methods such as yeast two-hybrid, Co-Immunoprecipitation (Co-IP), Bimolecular Fluorescence Complementation (BIFC), and Pull Down are required to confirm their interaction in the NF-κB pathway of *P. xylostella*. However, despite this, we have seen the precise “Lock-and-Key Interaction Model” between PxDorsal and PxCactus through high-quality three-dimensional structural simulations, which not only demonstrates the magic of biological interaction, but also provides a new understanding of the mechanism of interaction between these proteins and the regulation of the expression of antimicrobial peptides.

*Marsupenaeus japonicus* and *Plautia stali* have been shown to regulate the expression of antimicrobial peptides when challenged with bacteria [45,46]. When invaded by pathogenic microorganisms, Dorsal and Cactus, as NF-κB and IκB proteins, regulate the transcription of downstream antimicrobial peptides, promoting the production of antimicrobial peptides to resist and eliminate pathogens [1]. Spatio-temporal expression analysis showed that *PxDorsal* and *PxCactus* are expressed in all developmental stages of *P. xylostella*. However, it is very interesting that we found that following infection with Bt8010 for 12 h, the expression of *PxDorsal*, *PxCactus*, *PxGloverin*2, *PxMoricin*3, and *PxLysozyme*2 were significantly down-regulated. These findings suggest that PxDorsal and PxCactus may participate in regulating the expression of antimicrobial peptides in *P. xylostella*, and most interestingly, Bt can negatively regulate the Toll pathway of *P. xylostella*. Throughout the long process of evolution, there has been an interactive struggle between pathogens and their hosts. Hosts evolve immune systems to combat pathogens, while pathogens also evolve defensive mechanisms to suppress the host’s immune system. The inhibitory mechanism of Bt on the immune system of the *P. xylostella* when feeding under Bt, particularly regarding the Toll pathway, is a direction worth exploring in the future.

Silencing the *PxDorsal* gene significantly down-regulated the expression of antimicrobial peptide genes *PxGloverin*2 and *PxMoricin*3, indicating that *PxDorsal* primarily regulates these antimicrobial peptides. However, there is no effect on *PxCecropin*2 and *PxLysozyme*2, probably because *PxCecropin*2 and *PxLysozyme*2 are regulated by different transcription factors or immune pathways, such as the IMD pathway, JNK pathway or factors in other pathways. Previous studies have also shown that *Cactus* gene knockdown in *P. xylostella* affects antimicrobial peptide gene expression [33]. These findings confirm that PxDorsal and PxCactus interact to regulate the downstream antimicrobial peptides in the Toll pathway. Additionally, observations of *P. xylostella* larvae fed Bt8010 for 12 h after *PxDorsal* silencing showed a significant increase in epidermis melanization, which suggests that silencing *PxDorsal* may enhance susceptibility to Bt8010. This result is consistent with the observation that the knockdown of *SpDorsal* in *S. paramamosain* decreased its ability to defend against *Staphylococcus aureus* [13]. Melanization is primarily controlled by phenoloxidase (PO). Pro-phenoloxidase (PPO) is cleaved and activated by serine proteases to form PO, which then catalyzes tyrosine into quinones, thus triggering melanization [47,48,49]. In *Tenebrio molitor*, the Spätzle-processing enzyme (SPE) in the Toll pathway can participate in Spätzle processing, induce the production of antimicrobial peptides, and trigger a melanization response by activating PPO and SPH1 to form a melanization complex and play a crucial role in insect immune defense [50]. Our findings that the down-regulation of antimicrobial peptide immunity can lead to a novel phenotype of epidermis melanization phenomenon suggests the importance of Toll pathway-mediated antimicrobial peptide expression in host response to pathogenic bacterial infestation, as well as the multiplicity and complexity of insect immunity. Together, these studies confirm that the *PxDorsal* regulates the expression of *PxGloverin*2 and *PxMoricin*3, affecting the susceptibility of *P. xylostella* to Bt1080.

**Figure 6:**
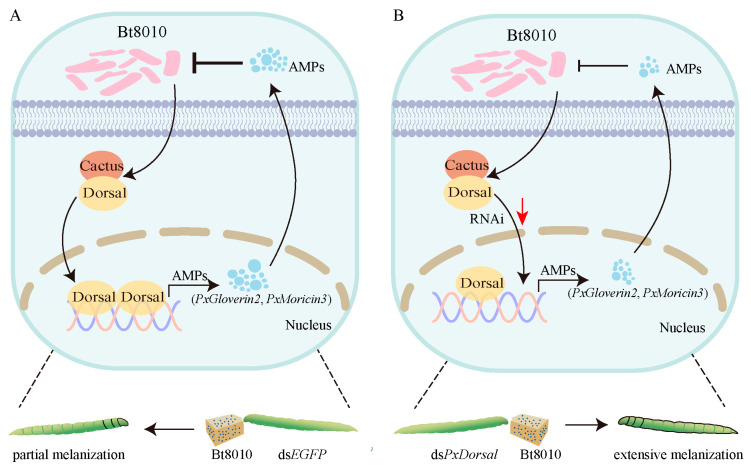
Model of NF-κB pathway regulating Bt susceptibility in *Plutella xylostella*. (**A**) PxDorsal and PxCactus interactively regulate the antimicrobial peptides PxGloverin2 and *PxMoricin*3 in *P. xylostella*, thereby inhibiting the proliferation of Bt and resulting in the phenomenon of partial epidermis melanization; (**B**) due to the down-regulation of the expression of *PxDorsal*, the expression of *PxGloverin*2 and *PxMoricin*3 were down-regulated, which increases the susceptibility of *P. xylostella* to Bt and causes an accelerated melanization of the epidermis.

This study, however, has some limitations; particularly, the inability to further investigate the functions of PxDorsal and PxCactus in *P. xylostella*. Since Dorsal and Cactus are transcription factors involved in innate immunity, and *PxDorsal* can participate in early embryonic development, we failed to obtain *PxDorsal* and *PxCactus* homozygous mutants by CRISPR/Cas9. In addition, only one *PxRPL*32 gene was used as an internal reference gene for RT-qPCR, and to ensure the reliability of RT-qPCR analysis results, studies can choose two genes to be used together as internal reference genes in the future. Additionally, the specific molecular mechanism of the interaction of PxDorsal and PxCactus remains to be explored by experiments. In addition to this, the susceptibility of the *P. xylostella* to Bt may be related to a variety of factors, including the activity and efficiency of its innate immune system. The interaction between PxDorsal and PxCactus affects the expression and function of downstream antimicrobial peptides, which could indirectly affect the susceptibility of *P. xylostella* to Bt. However, the specific mechanisms and extent of this relationship are currently unclear and require further exploration through future molecular biology and genetic studies. Despite these limitations, our study has confirmed that PxDorsal and PxCactus exhibit the structural characteristics of NF-κB and IκB factors, and they can interact with each other. Additionally, PxDorsal functions as an NF-κB factor to regulate the expression of *PxGloverin*2 and *PxMoricin*3, thereby protecting against Bt8010 infection (Figure 6). This study provides a new insight for further study on the susceptibility of *P. xylostella* to invasive pathogens through the Toll pathway and also provides ideas for promoting the screening of effective biological control targets for *P. xylostella* from an immunological perspective.

## Data Availability

The data for this study can be requested by contacting the author.

## Supplementary Materials

The following supporting information can be downloaded at: https://www.mdpi.com/article/10.3390/insects16020163/s1, Figure S1: The standard curve for an amplification efficiency for RT-qPCR primers and the dsRNA interference efficiency; Figure S2: Sequence analysis of PxDorsal; Figure S3: Sequence analysis of PxCactus; Figure S4: Multiple sequence alignment of PxDorsal; Figure S5: Multiple sequence alignment of PxCactus; Figure S6: Conserved domains in PxDorsal and PxCactus models; Figure S7: Docking model surface interaction of amino acids; Table S1: Primer sequences of RT-qPCR; Table S2: Primer sequences for RNAi; Table S3: Amino acid composition of PxDorsal; Table S4: Amino acid composition of PxCactus; Table S5: The PxDorsal and PxCactus models were detected by PROCHECK; Table S6: Amino acids that form hydrogen bonds on the protein binding surface.

## References

[1] Hoffmann J.A. (2003). The immune response of *Drosophila*. Nature.

[2] Ghosh S., May M.J., Kopp E.B. (1998). NF-κB and Rel proteins: Evolutionarily conserved mediators of immune responses. Annu. Rev. Immunol..

[3] Hetru C., Hoffmann J.A. (2009). NF-κB in the immune response of *Drosophila*. Cold Spring Harb. Perspect. Biol..

[4] Chowdhury M., Zhang J., Xu X.X., He Z., Lu Y., Liu X.S., Wang Y.F., Yu X.Q. (2019). An in vitro study of NF-κB factors cooperatively in regulation of *Drosophila melanogaster* antimicrobial peptide genes. Dev. Comp. Immunol..

[5] Geisler R., Bergmann A., Hiromi Y., Nüsslein-Volhard C. (1992). Cactus, a gene involved in dorsoventral pattern formation of *Drosophila*, is related to the IκB gene family of vertebrates. Cell.

[6] Luque I., Zong W.X., Chen C., Gélinas C. (2000). N-terminal determinants of IκBα necessary for the cytoplasmic regulation of c-Rel. Oncogene.

[7] Furukawa S., Tanaka H., Ishibashi J., Imanishi S., Yamakawa M. (2009). Functional characterization of a cactus homolog from the silkworm *Bombyx mori*. Biosci. Biotechnol. Biochem..

[8] Wang D., Li F., Li S., Chi Y., Wen R., Feng N., Xiang J. (2013). An IκB homologue (*FcCactus*) in Chinese shrimp *Fenneropenaeus chinensis*. Dev. Comp. Immunol..

[9] Li C., Chen Y.X., Zhang S., Lu L., Chen Y.H., Chai J., Weng S., Chen Y.G., He J., Xu X. (2012). Identification, characterization, and function analysis of the Cactus gene from *Litopenaeus vannamei*. PLoS ONE.

[10] Whalen A.M., Steward R. (1993). Dissociation of the dorsal-cactus complex and phosphorylation of the dorsal protein correlate with the nuclear localization of dorsal. J. Cell Biol..

[11] Baeuerle P.A., Henkel T. (1994). Function and activation of NF-κB in the immune system. Annu. Rev. Immunol..

[12] Zhong X., Rao X.J., Yi H.Y., Lin X.Y., Huang X.H., Yu X.Q. (2016). Co-expression of Dorsal and Rel2 negatively regulates antimicrobial peptide expression in the tobacco hornworm *Manduca sexta*. Sci. Rep..

[13] Deng H., Hu L., Li J., Yan W., Song E., Kuang M., Liu S., He J., Weng S. (2020). The NF-κB family member dorsal plays a role in immune response against Gram-positive bacterial infection in mud crab (*Scylla paramamosain*). Dev. Comp. Immunol..

[14] Ding D., Sun X.J., Yan M., Chen Q., Gao L., Kang C.J. (2022). The ECSIT Mediated Toll3-Dorsal-ALFs Pathway Inhibits Bacterial Amplification in Kuruma Shrimp. Front. Immunol..

[15] Nishide Y., Nagamine K., Kageyama D., Moriyama M., Futahashi R., Fukatsu T. (2022). A new antimicrobial peptide, Pentatomicin, from the stinkbug *Plautia stali*. Sci. Rep..

[16] Lemaitre B., Reichhart J.M., Hoffmann J.A. (1997). *Drosophila* host defense differential induction of antimicrobial peptide genes after infection by various classes ofmicroorganisms. Proc. Natl. Acad. Sci. USA.

[17] Levashina E.A., Ohresser S., Lemaitre B., Imler J.L. (1998). Two distinct pathways can control expression of the gene encoding the *Drosophila* antimicrobial peptide metchnikowin. J. Mol. Biol..

[18] Manfruelli P., Reichhart J., Steward R., Hoffmann J.A., Lemaitre B. (1999). A mosaic analysis in *Drosophila* fat body cells of the control of antimicrobial peptide genes by the Rel proteins Dorsal and DIF. EMBO J..

[19] Lemaitre B., Kromer-Metzger E., Michaut L., Nicolas E., Meister M., Georgel P., Reichhart J.M., Hoffmann J.A. (1995). A recessive mutation, immune deficiency (*imd*), defines two distinct control pathways in the *Drosophila* host defense. Proc. Natl. Acad. Sci. USA.

[20] Lindsay S.A., Wasserman S.A. (2014). Conventional and non-conventional *Drosophila* Toll signaling. Dev. Comp. Immunol..

[21] Xia X., Yu L., Xue M., Yu X., Vasseur L., Gurr G.M., Baxter S.W., Lin H., Lin J., You M. (2015). Genome-wide characterization and expression profiling of immune genes in the diamondback moth, *Plutella xylostella* (L.). Sci. Rep..

[22] Lin J., Xia X., Yu X.Q., Shen J., Li Y., Lin H., Tang S., Vasseur L., You M. (2018). Gene expression profiling provides insights into the immune mechanism of *Plutella xylostella* midgut to microbial infection. Gene.

[23] Furlong M.J., Wright D.J., Dosdall L.M. (2013). Diamondback moth ecology and management: Problems, progress, and prospects. Annu. Rev. Entomol..

[24] Guo Z., Sun D., Kang S., Zhou J., Gong L., Qin J., Guo L., Zhu L., Bai Y., Luo L. (2019). CRISPR/Cas9-mediated knockout of both the *PxABCC2* and *PxABCC3* genes confers high-level resistance to *Bacillus thuringiensis* Cry1Ac toxin in the diamondback moth, *Plutella xylostella* (L.). Insect Biochem. Mol. Biol..

[25] Grizanova E.V., Dubovskiy I.M., Whitten M.M., Glupov V.V. (2014). Contributions of cellular and humoral immunity of *Galleria mellonella* larvae in defence against oral infection by *Bacillus thuringiensis*. J. Invertebr. Pathol..

[26] Contreras E., Benito-Jardon M., Lopez-Galiano M.J., Real M.D., Rausell C. (2015). *Tribolium castaneum* immune defense genes are differentially expressed in response to *Bacillus thuringiensis* toxins sharing common receptor molecules and exhibiting disparate toxicity. Dev. Comp. Immunol..

[27] Song F., Chen C., Wu S., Shao E., Li M., Guan X., Huang Z. (2016). Transcriptional profiling analysis of *Spodoptera litura* larvae challenged with Vip3Aa toxin and possible involvement of trypsin in the toxin activation. Sci. Rep..

[28] Wu G., Yi Y. (2018). Transcriptome analysis of differentially expressed genes involved in innate immunity following *Bacillus thuringiensis* challenge in *Bombyx mori* larvae. Mol. Immunol..

[29] Dubovskiy I.M., Grizanova E.V., Whitten M.M., Mukherjee K., Greig C., Alikina T., Kabilov M., Vilcinskas A., Glupov V.V., Butt T.M. (2016). Immuno-physiological adaptations confer wax moth Galleria mellonella resistance to *Bacillus thuringiensis*. Virulence.

[30] Wei J., Yang S., Chen L., Liu X., Du M., An S., Liang G. (2018). Transcriptomic responses to different Cry1Ac selection stresses in *Helicoverpa armigera*. Front. Physiol..

[31] Lu K., Gu Y., Liu X., Lin Y., Yu X.Q. (2017). Possible insecticidal mechanisms mediated by immune response related Cry-binding proteins in the midgut juice of *Plutella xylostella* and *Spodoptera exigua*. J. Agric. Food Chem..

[32] Xu X.X., Zhang Y.Q., Freed S., Yu J., Gao Y.F., Wang S., Ouyang L.N., Ju W.Y., Jin F.L. (2016). An anionic defensin from *Plutella xylostella* with potential activity against *Bacillus thuringiensis*. Bull. Entomol. Res..

[33] Gao Y.F. (2017). Comparative Identification of Immune Related Proteins at Different Development Stages of *Plutella xylostella* and Functional Study on Cactus Gene. Master’s Thesis.

[34] Valanne S., Wang J.H., Ramet M. (2011). The *Drosophila* Toll signaling pathway. J. Immunol..

[35] Stokes B.A., Yadav S., Shokal U., Smith L.C., Eleftherianos I. (2015). Bacterial and fungal pattern recognition receptors in homologous innate signaling pathways of insects and mammals. Front. Microbiol..

[36] Lin J., Yu X.Q., Wang Q., Tao X., Li J., Zhang S., Xia X., You M. (2020). Immune responses to *Bacillus thuringiensis* in the midgut of the diamondback moth, *Plutella xylostella*. Dev. Comp. Immunol..

[37] Saitou N., Nei M. (1987). The neighbor-joining method a new method for reconstructing phylogenetic trees. Mol. Biol. Evol..

[38] Tamura K., Peterson D., Peterson N., Stecher G., Nei M., Kumar S. (2011). MEGA5: Molecular evolutionary genetics analysis using maximum likelihood, evolutionary distance, and maximum parsimony methods. Mol. Biol. Evol..

[39] Livak K.J., Schmittgen T.D. (2001). Analysis of relative gene expression data using real-time quantitative PCR and the 2^−ΔΔCT^ Method. Methods.

[40] Whyard S., Singh A.D., Wong S. (2009). Ingested double-stranded RNAs can act as species-specific insecticides. Insect Biochem. Mol. Biol..

[41] Hatada E.N., Nieters A., Wulczyn F.G., Naumann M., Meyer R., Nucifora G., McKeithan T.W., Scheidereit C. (1992). The ankyrin repeat domains of the NF-κB precursor p105 and the protooncogene *bc1-3* act as specific inhibitors of NF-κB DNA binding. Proc. Natl. Acad. Sci. USA.

[42] Xu X., Yuan J., Yang L., Weng S., He J., Zuo H. (2016). The Dorsal/miR-1959/Cactus feedback loop facilitates the infection of WSSV in *Litopenaeus vannamei*. Fish Shellfish Immunol..

[43] Leclerc V., Reichhart J.-M. (2004). The immune response of *Drosophila melanogaster*. Immunol. Rev..

[44] Lu X.M., Ye G.Y. (2006). NF-κB signaling in insects. Chin. J. Cell Biol..

[45] Sun J.J., Xu S., He Z.H., Shi X.Z., Zhao X.F., Wang J.X. (2017). Activation of Toll Pathway Is Different between Kuruma Shrimp and *Drosophila*. Front. Immunol..

[46] Nishide Y., Kageyama D., Yokoi K., Jouraku A., Tanaka H., Futahashi R., Fukatsu T. (2019). Functional crosstalk across IMD and Toll pathways: Insight into the evolution of incomplete immune cascades. Proc. Biol. Sci..

[47] Jiang H., Wang Y., Kanost M.R. (1998). Pro-phenol oxidase activating proteinase from an insect, *Manduca sexta*: A bacteria-inducible protein similar to *Drosophila* easter. Proc. Natl. Acad. Sci. USA.

[48] Cerenius L., Söderhäll K. (2004). The prophenoloxidase-activating system in invertebrates. Immunol. Rev..

[49] Barillas-Mury C. (2007). CLIP proteases and *Plasmodium* melanization in *Anopheles gambiae*. Trends Parasitol..

[50] Kan H., Kim C.H., Kwon H.M., Park J.W., Roh K.B., Lee H., Park B.J., Zhang R., Zhang J., Soderhall K. (2008). Molecular control of phenoloxidase-induced melanin synthesis in an insect. J. Biol. Chem..

