# Supplementary material for: PxDorsal Regulates the Expression of Antimicrobial Peptides and Affects the Bt Susceptibility of Plutella xylostella
Source: Insects. 2025 Feb 5;16(2):163. doi: 10.3390/insects16020163 (PMC11855926; doi:10.3390/insects16020163)
Supplement: Supplementary file 1 [file insects-16-00163-s001.zip › insects-3430083-supplementary.pdf]

Supplementary Information

PxDorsal Regulates the Expression of Antimicrobial Peptides and Affects the Bt Susceptibility of *Plutella xylostella*

Yan Sun[1,2,3,4†], Haoqi Wen[1,2,3,4†], Wenrui Xue[1,2,3,4†], Xiaofeng Xia[1,2,3,4*]

[1]State Key Laboratory of Agricultural and Forestry Biosecurity, Institute of Applied Ecology, Fujian Agriculture and Forestry University, Fuzhou 350002, China;

[2]Key Laboratory of Integrated Pest Management for Fujian-Taiwan Crops, Ministry of Agriculture and Rural Affairs, Fuzhou 350002, China;

[3]Joint International Research Laboratory of Ecological Pest Control, Ministry of Education, Fuzhou 350002, China;

[4]Youxi-Yangzhong Vegetable Pest Prevention and Control, Fujian Observation and Research Station, Fuzhou 350002, China.

[*]Correspondence to X.X. (Email: xiaofengxia@fafu.edu.cn).

[†]These authors contributed equally to this work.

This file includes:

Supplementary Figures S1~S7

Supplementary Tables S1~S6

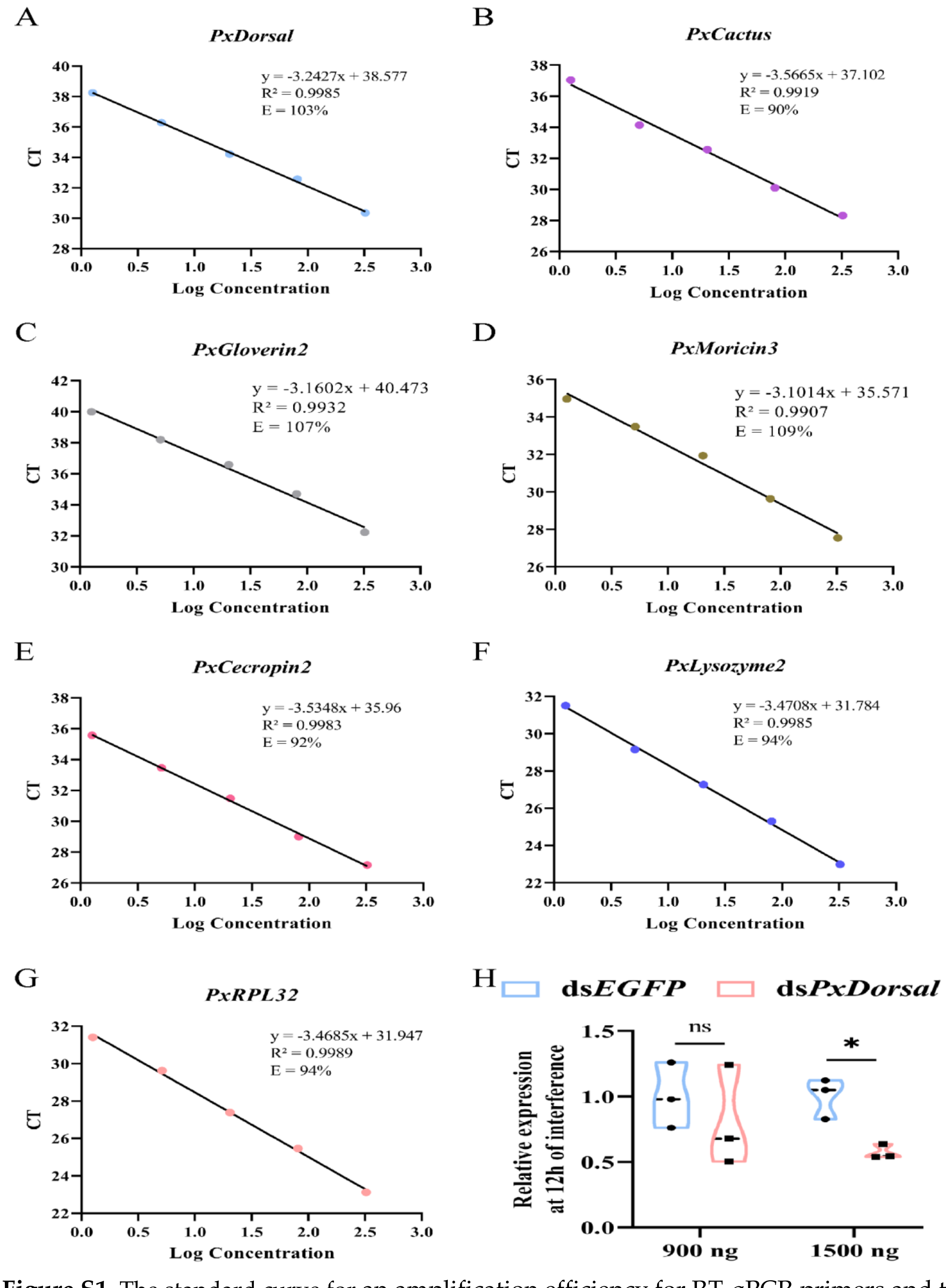

Figure S1. The standard curve for an amplification efficiency for RT-qPCR primers and the dsRNA interference efficiency. (A-G) An amplification efficiency ranging from 90%-110% is generally regarded as ideal, and the amplification efficiencies of the aforementioned primers all fall within 90%-110% range, indicating that these primers are suitable for RT-qPCR experiments; (H) Quantify the interference efficiency of 900 ng and 1500 ng dsRNA, injection of 1500 ng ds*PxDorsal* significantly silence the *PxDorsal* gene.

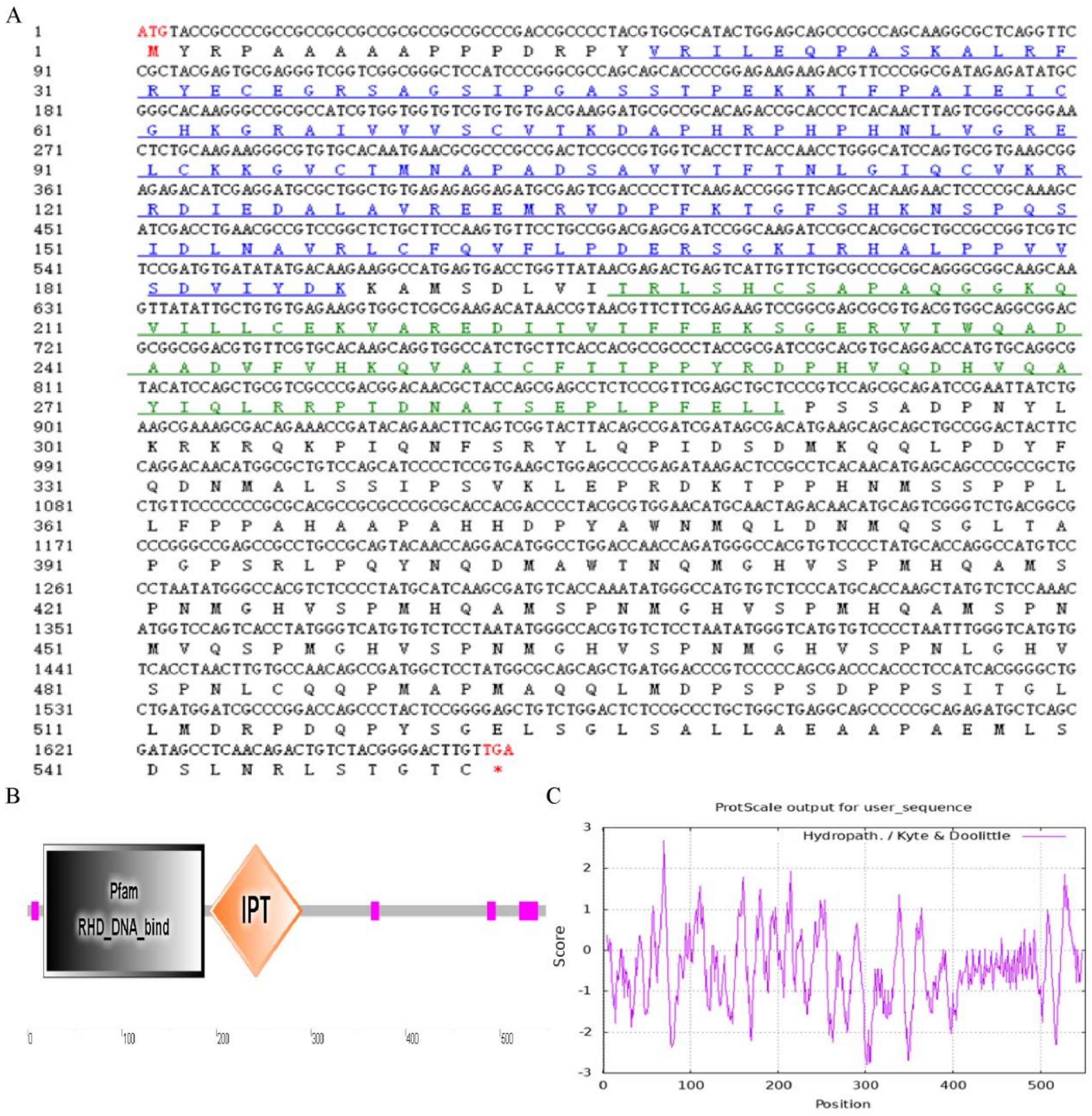

Figure S2. Sequence analysis of PxDorsal. (A) The open reading frame (ORF) sequence and encoded amino acid sequence of PxDorsal, the start and stop codons highlighted in red, and distinct domains indicated by blue and green sequences; (B) Protein structure prediction of PxDorsal; (C) Hydrophilicity/hydrophobicity prediction of PxDorsal.

A

```
1     ATGAGTTTCAAGAAGGATTTCGACACCTCAAAGAAGATCCAGGAGGATGAAAACACAGACTCTGGGTTCCTATCTGGGCCGATAAGTGAG
1      M  S  F  K  K  D  F  D  T  S  K  K  I  Q  E  D  E  N  T  D  S  G  F  L  S  G  P  I  S  E
91    CAGCTGACCTCGGAAGATTGTGATTTAGCGGAGGAAAGTGAGCGTGCTCGCAGCAGGCTTAGTGAGGAAGATCCTGAGCCTGAGCTGCAG
31     Q  L  T  S  E  D  C  D  L  A  E  E  S  E  R  A  R  S  R  L  S  E  E  D  P  E  P  E  L  Q
181   TTGGACAGTGGGCTGGACCTCTCGGAGTGTCTGTCGAGTGTTAAGCTTAGTGATAGTGCAGTGTACACACCCACCTCGCAGACCACCCCC
61     L  D  S  G  L  D  L  S  E  C  L  S  S  V  K  L  S  D  S  A  V  Y  T  P  T  S  Q  T  T  P
271   ACAGTCACTATAGGTGATGAGAAAACTCATGACATCCCACCCCTCGCCATCCTGTTCCAGCAGGATGACGATGGAGACACACAACTACAC
91     T  V  T  I  G  D  E  K  T  H  D  I  P  P  L  A  I  L  F  Q  Q  D  D  D  G  D  T  Q  L  H
361   ATTGCAGCGGTACATGGGTGCGAAAAATCAGTAGGAACATTAGTAAGAGTTTGCCCTGACAAAGATTGGCTAAATGTACCAAATGACTTT
121    I  A  A  V  H  G  C  E  K  S  V  G  T  L  V  R  V  C  P  D  K  D  W  L  N  V  P  N  D  F
451   GGACAGACCGCCTTACACTTAGCAGCCATGAGTGGGCATGCAGTAGTCACACGCATGCTGGTGATGGCCGGTGCATCTCTTGGCATTCGA
151    G  Q  T  A  L  H  L  A  A  M  S  G  H  A  V  V  T  R  M  L  V  M  A  G  A  S  L  G  I  R
541   GACCTTGTTGGCAACACACCTTTACATGTGGCAGCCGCAGCGGGCTACGTCGGCTGTCTCCAAGCTTTACTGGCTCCTGCTCCAGAACAA
181    D  L  V  G  N  T  P  L  H  V  A  A  A  A  G  Y  V  G  C  L  Q  A  L  L  A  P  A  P  E  Q
631   CAGCAGAGAAGGCTATCATCCACGTTGAACCAGAAAAATTACAATGGTCAAACGTGCGTCCATGTGGCTGCGATGGCCGGCCACGTCGAC
211    Q  Q  R  R  L  S  S  T  L  N  Q  K  N  Y  N  G  Q  T  C  V  H  V  A  A  M  A  G  H  V  D
721   GCGCTGCAGACATTGGTCTATTACGGAGCTAACATCAATGCTGCGGAGGGTCTATGCGGGTGGACACCGCTACACGTGGCGGCTGCGCGA
241    A  L  Q  T  L  V  Y  Y  G  A  N  I  N  A  A  E  G  L  C  G  W  T  P  L  H  V  A  A  A  R
811   GGGGACGTCGACACGGCCCGCTACTTGCTCGAGAAGTGCGCGGGCGTCGACCCCTCTGCCCTGGACTACGCCGGTCGTACGGCCAGGAAG
271    G  D  V  D  T  A  R  Y  L  L  E  K  C  A  G  V  D  P  S  A  L  D  Y  A  G  R  T  A  R  K
901   CTAGCAATGAAGAATAAAGCGGCTGCCCTGTTTGACGGCAGTGAGGGCAGCGAGGAGGAGGATAGTGATAGTGAGGATGAGATGCTTCTG
301    L  A  M  K  N  K  A  A  A  L  F  D  G  S  E  G  S  E  E  E  D  S  D  S  E  D  E  M  L  L
991   GAAAGCGACCAGAGTCTGTTCGACCGGATCCGTGACGGTATGAACGCCATCAACGTCGCCTGA
331    E  S  D  Q  S  L  F  D  R  I  R  D  G  M  N  A  I  N  V  A  *
```

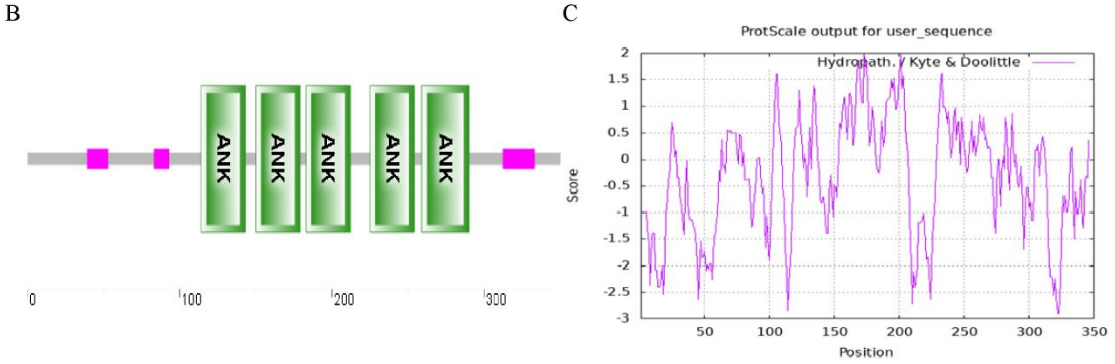

Figure S3. Sequence analysis of PxCactus. (A) The ORF sequence and encoded amino acid sequence of PxCactus, the start and stop codons highlighted in red, and distinct domains indicated by blue and green sequences; (B) Protein structure prediction of PxCactus; (C) Hydrophilicity/hydrophobicity prediction of PxCactus.

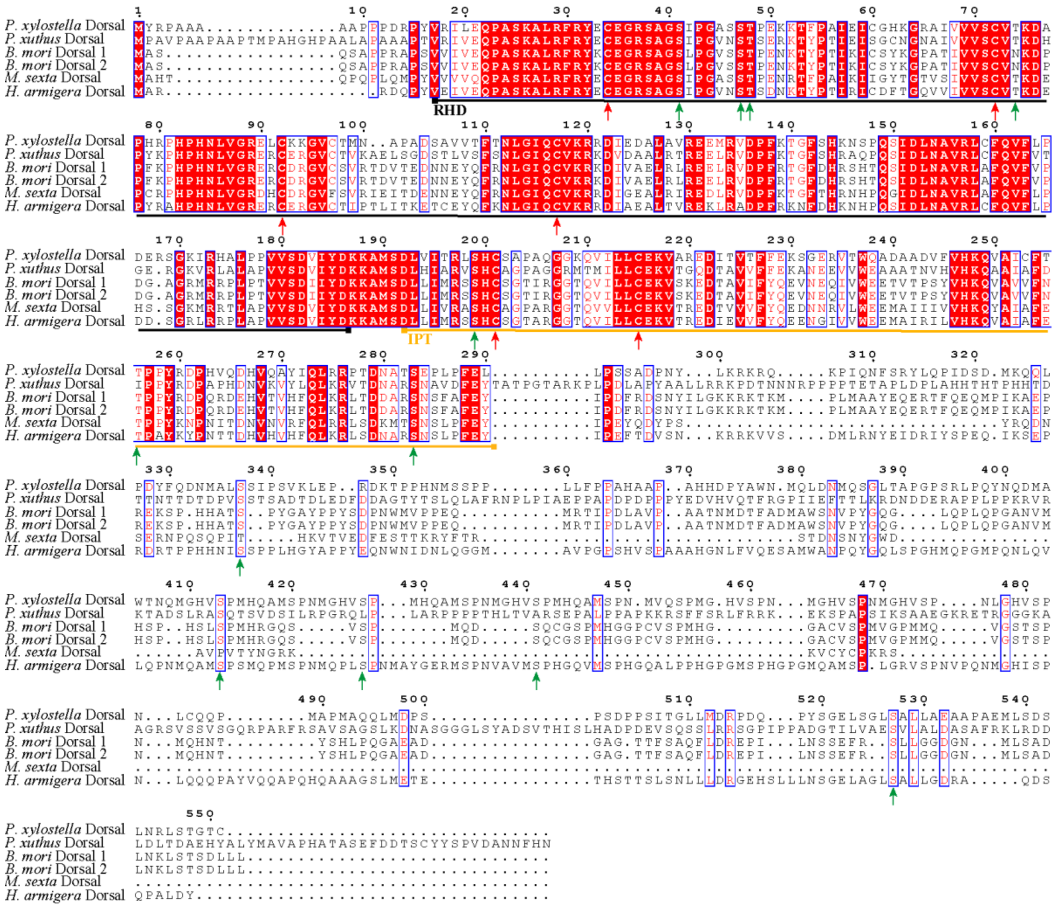

Figure S4. Multiple sequence alignment of PxDorsal. The black underlined sequence is the RHD domain, the orange underline sequence is an IPT domain; conserved Cys residues and P-phosphorylation sites are indicated by red and green arrows, respectively. *P. xylostella* Dorsal: *Plutella xylostella* Dorsal (OQ439920.1); *P. xuthus* Dorsal: *Papilio xuthus* Dorsal (KPJ05518.1); *B. mori* Dorsal 1: *Bombyx mori* Dorsal 1 (NP_001166296.1); *B. mori* Dorsal 2: *Bombyx mori* Dorsal 2 (NP_001036896.1); *M. sexta* Dorsal: *Manduca sexta* Dorsal (ADK39025.1); *H.armigera* Dorsal: *Helicoverpa armigera* Dorsal (AEO51736.1).

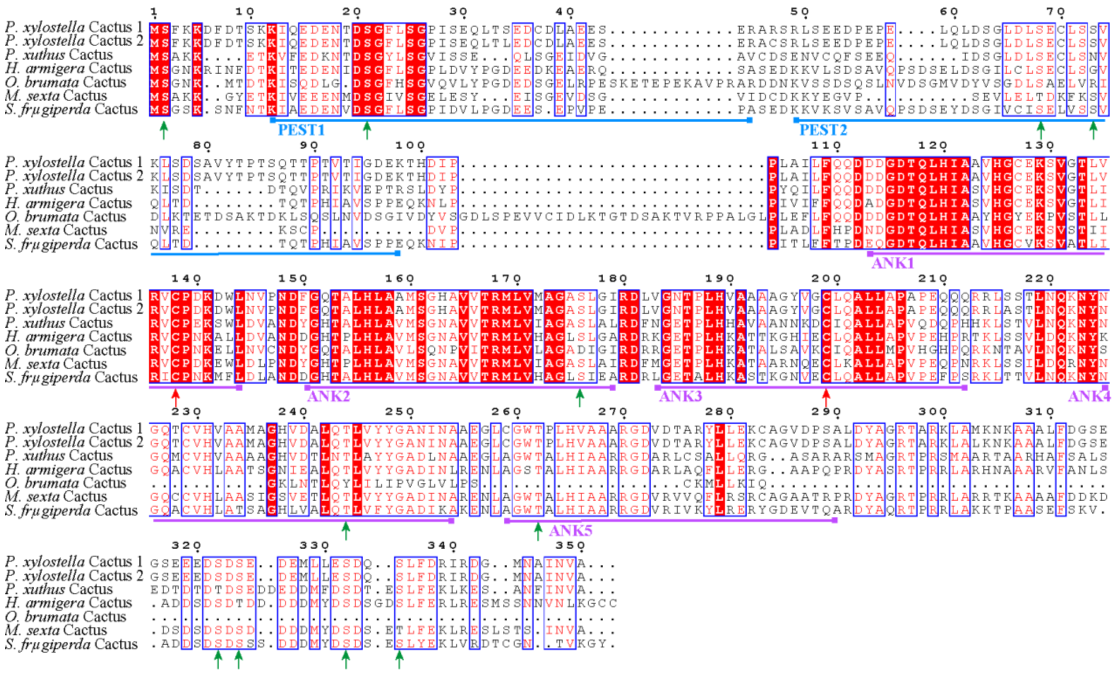

Figure S5. Multiple sequence alignment of PxCactus. The blue underline sequence is a PEST domain, and the purple underlined sequences are ANKs repeat sequence; conserved Cys residues and P-phosphorylation sites are indicated by red and green arrows, respectively. *P. xylostella* Cactus 1: *Plutella xylostella* Cactus 1 (OQ439953.1); *P. xylostella* Cactus 2: *Plutella xylostella* Cactus 2 (AWD92943.1); *P. xuthus* Cactus: *Papilio Xuthus* Cactus (KPI96888.1); *H. armigera* Cactus: *Helicoverpa armigera* Cactus (XP_021191206.2); *O. brumata* Cactus: *Operophtera brumata* Cactus (KOB67992.1); *M. sexta* Cactus: *Manduca sexta* Cactus (XP_030020657.1); *S. frμgiperda* Cactus: *Spodoptera frμgiperda* Cactus (XP_035433521.1).

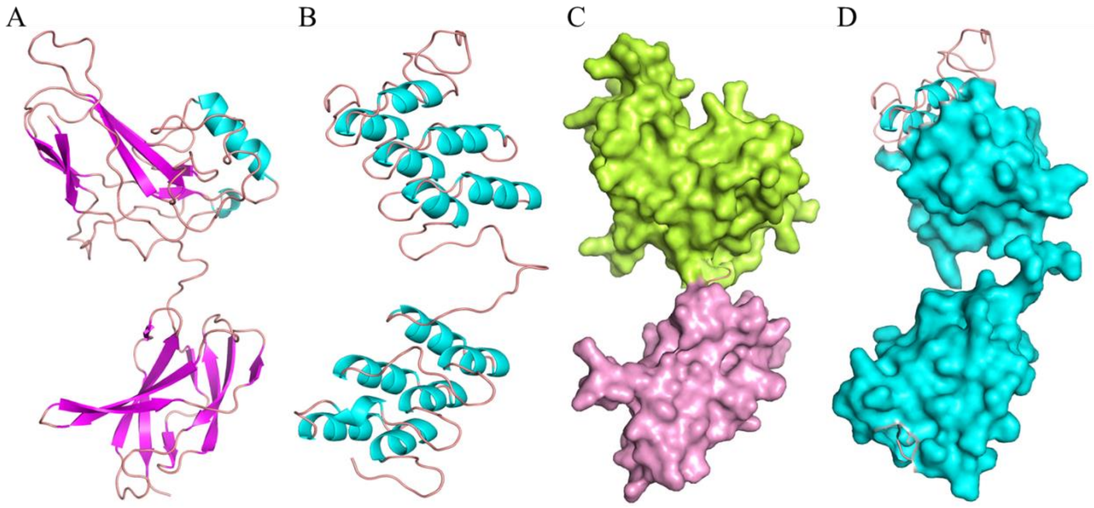

Figure S6. Conserved domains in PxDorsal and PxCactus models. (A) The cartoon diagram of PxDorsal model; (B) The cartoon diagram of PxCactus model; (C) The surface diagram of PxDorsal model; (D) The surface diagram of the PxCactus model. The yellow-green structure is the RHD domain, the pink structure is the IPT domain, and the blue structure is the ANKs domain.

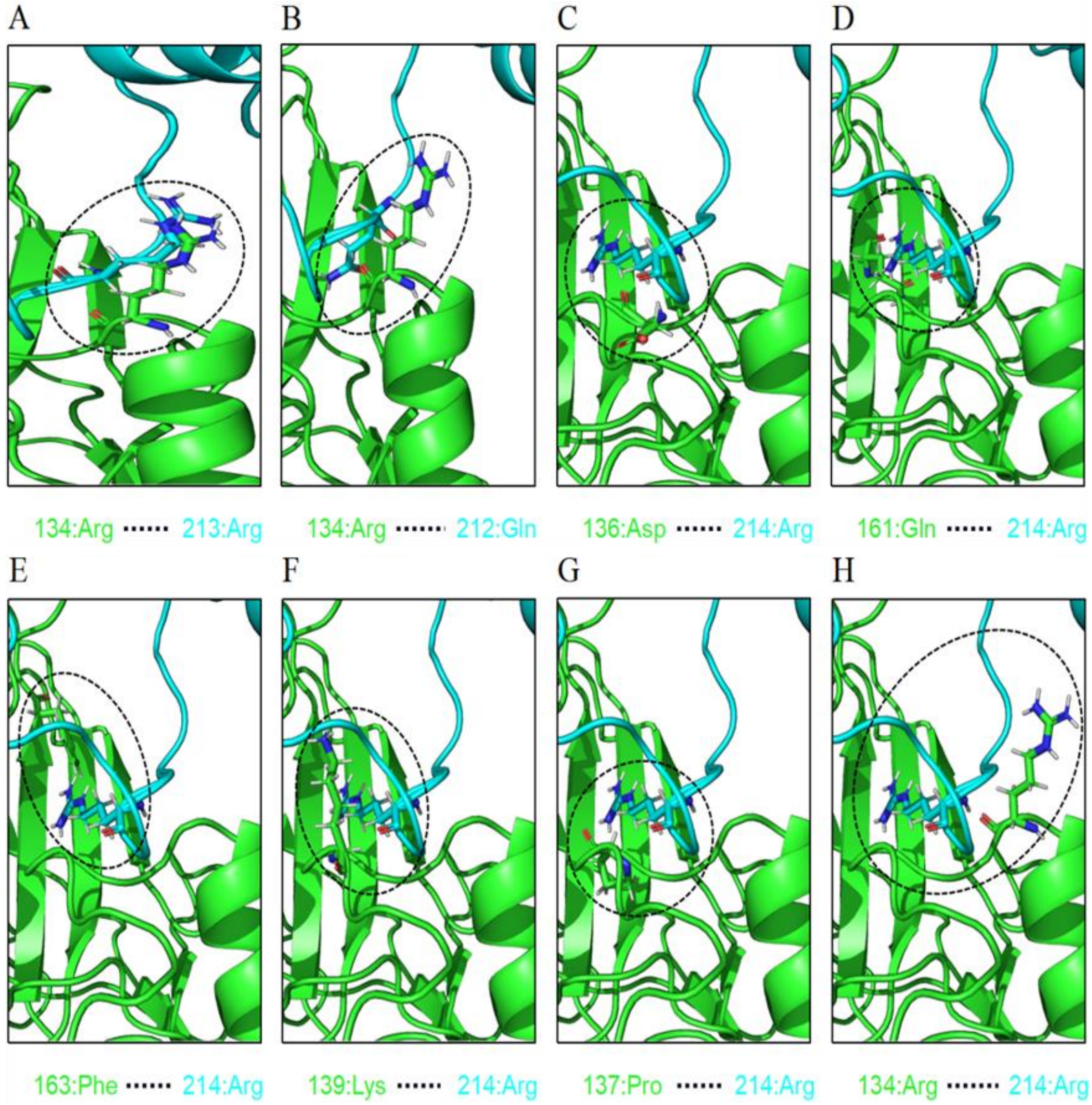

Figure S7. Docking model surface interaction of amino acids. (A-H) The interaction interface between PxDorsal and PxCactus, PxDorsal and its amino acids are shown in green, and PxCactus and its amino acids are shown in blue, and "-----" at the bottom of the figure indicates hydrogen bonds through which amino acid residues, and the oval indicates the region of hydrogen bond binding.

Table S1. Primer sequences of RT-qPCR

Primer name	Sequence (5'-3')
PxDorsal-F	AGCGAAAGCGACAGAAACCGATAC
PxDorsal-R	CGCCATGTTGTCCTGGAAGTAGTC
PxCactus-F	TCGACACCTCAAAGAAGATCCAG
PxCactus-R	TCACTTTCCTCCGCTAAATCACA
PxGloverin2-F	GCCGGGCTATTCTTACAAGC
PxGloverin2-R	CCCGTAGTCTGGTCTCCCAT
PxMoricin3-F	GAAGGGCGGCCATGTCATTA
PxMoricin3-R	TACCCCTGGTTCCTGTTCCG
PxCecropin2-F	TTCGTGTTCGTGGCTGTTTT
PxCecropin2-R	GGCCAACTCTTTCCAATTTCTT
PxLyszoimes2-F	AGTTGATAACTGACGACATCACGAA
PxLyszoimes2-R	GGCACTTGTTCTTCCATCCATAC
RPL32-F	CAATCAGGCCAATTTACCGC
RPL32-R	CTGGGTTTACGCCAGTTACG

Table S2. Primer sequences for RNAi

Primer name	Sequence (5’-3’)
ds*PxDorsal*-F	TAATACGACTCACTATAGGGGATCCGCCACGCGCTG
ds*PxDorsal*-R	TAATACGACTCACTATAGGGAGCTGGATGTACGCCTG
ds*EGFP*-F	TAATACGACTCACTATAGGGCCACAAGTTCAGCGTGTCCG
ds*EGFP*-R	TAATACGACTCACTATAGGGTTCAGCTCGATGCGGTTC

The underlined is the T7 polymerase promoter sequence.

Table S3. Amino acids composition of PxDorsal

Amino acid residue	Number	Proportion (%)
Ala (A)	48	8.7
Arg (R)	29	5.3
Asn (N)	22	4.0
Asp (D)	31	5.6
Cys (C)	12	2.2
Gln (Q)	31	5.6
Glu (E)	20	3.6
Gly (G)	26	4.7
His (H)	24	4.4
Ile (I)	19	3.4
Leu (L)	40	7.3
Lys (K)	23	4.2
Met (M)	28	5.1
Phe (F)	15	2.7
Pro (P)	63	11.4
Ser (S)	48	8.7
Thr (T)	21	3.8
Trp (W)	3	0.5
Tyr (Y)	12	2.2
Val (V)	36	6.5

Table S4. Amino acids composition of PxCactus

Amino acid residue	Number	Proportion (%)
Ala (A)	40	11.4
Arg (R)	14	4
Asn (N)	12	3.4
Asp (D)	32	9.1
Cys (C)	8	2.3
Gln (Q)	16	4.6
Glu (E)	24	6.9
Gly (G)	25	7.1
His (H)	9	2.6
Ile (I)	10	2.9
Leu (L)	38	10.9
Lys (K)	13	3.7
Met (M)	8	2.3
Phe (F)	7	2.0
Pro (P)	14	4.0
Ser (S)	28	8.0
Thr (T)	21	6.0
Trp (W)	2	0.6
Tyr (Y)	7	2.0
Val (V)	22	6.3

Table S5. The PxDorsal and PxCactus models were detected by PROCHECK

Regions	PxDorsal	PxCactus
The most favoured regions	79.8%	85.8%
The additional allowed regions	16.9%	11.8%
The generously allowed regions	2.1%	1.9%
The disallowed regions	1.2%	0.5%

The PROCHECK results showed that the disallowed region were less than 10% and the protein conformation was reasonable. The higher the proportion of the most favoured regions, the better the quality of the model, and the higher the credibility of the molecular docking results.

Table S6. Amino acids that form hydrogen bonds on the protein binding surface

Receptors-Ligands	Interacting-Amino acids	Hydrogen bond distance (Å)
PxDorsal-134: Arg	PxCactus-213: Arg	0.6 Å
	PxCactus-212: Gln	1.8 Å
PxCactus-214: Arg	PxDorsal-136: Asp	1.8 Å
	PxDorsal-161: Gln	2.1 Å
	PxDorsal-163: Phe	2.3 Å
	PxDorsal-139: Lys	2.7 Å
	PxDorsal-137: Pro	2.8 Å
	PxDorsal-134: Arg	3.2 Å

In molecular docking, the two can be each other's receptor and ligand. There are many amino groups in the side chain of arginine that are easy to form hydrogen bonds, and the hydrogen bonds between the receptor arginine and other amino acids of the ligand are listed. The smaller of the hydrogen bond distance, the stronger the interaction.
